# Spinal Interleukin-1β Inhibits Astrocyte Cytochrome P450c17 Expression Which Controls the Development of Mechanical Allodynia in a Mouse Model of Neuropathic Pain

**DOI:** 10.3389/fnmol.2019.00153

**Published:** 2019-06-20

**Authors:** Sheu-Ran Choi, Ho-Jae Han, Alvin J. Beitz, Jang-Hern Lee

**Affiliations:** ^1^Department of Veterinary Physiology, BK21 PLUS Program for Creative Veterinary Science Research, Research Institute for Veterinary Science, College of Veterinary Medicine, Seoul National University, Seoul, South Korea; ^2^Department of Veterinary and Biomedical Sciences, College of Veterinary Medicine, University of Minnesota, Saint Paul, MN, United States

**Keywords:** interleukin-1β, interleukin-1 receptor type 1, cytochrome P450c17, astrocytes, mechanical allodynia, neuropathic pain

## Abstract

We have recently demonstrated that sciatic nerve injury increases the expression of spinal cytochrome P450c17, a key neurosteroidogenic enzyme, which plays a critical role in the development of peripheral neuropathic pain. However, the modulatory mechanisms responsible for the expression of spinal P450c17 have yet to be examined. Here we investigated the possible involvement of interleukin-1β (IL-1β) in altering P450c17 expression during the induction phase of neuropathic pain. Neuropathic pain was produced by chronic constriction injury (CCI) of the right sciatic nerve in mice and mechanical allodynia was evaluated in the hind paws using a von-Frey filament (0.16 g). Western blotting and immunohistochemistry were performed to assess the expression of spinal IL-1β, interleukin-1 receptor type 1 (IL-1R1), P450c17, and GFAP. Spinal IL-1β was significantly increased on day 1 post-surgery and its receptor, IL-1R1 was expressed in GFAP-positive astrocytes. Intrathecal administration of the recombinant interleukin-1 receptor antagonist (IL-1ra, 20 ng) on days 0 and 1 post-surgery enhanced GFAP expression on day 1 post-surgery and induced an early increase in P450c17 expression in astrocytes, but not in neurons. Administration of IL-1β (10 ng) on days 0 and 1 post-surgery blocked the enhancement of both spinal P450c17 and GFAP expression induced by IL-1ra (20 ng) administration. Intrathecal administration of IL-1ra (20 ng) on days 0 to 3 post-surgery also facilitated the CCI-induced development of mechanical allodynia, and this early developed pain was dose-dependently attenuated by the administration of the P450c17 inhibitor, ketoconazole (1, 3, or 10 nmol) or the astrocyte metabolic inhibitor, fluorocitrate (0.01, 0.03, or 0.1 nmol). These results demonstrate that early increases in spinal IL-1β temporally inhibit astrocyte P450c17 expression and astrocyte activation ultimately controlling the development of mechanical allodynia induced by peripheral nerve injury. These findings imply that spinal IL-1β plays an important role as an early, but transient, control mechanism in the development of peripheral neuropathic pain via the inhibition of astrocyte P450c17 expression and astrocyte activation.

## Introduction

Neuropathic pain develops following injury to the nervous system and is often characterized by allodynia (the sensation of pain to non-noxious stimuli) and hyperalgesia (increased pain to a noxious stimuli) (Woolf and Mannion, 1999). The development of neuropathic pain involves a variety of pathophysiological changes in the nervous system, which are represented by peripheral sensitization (increased sensitivity of the nociceptor terminal) and central sensitization (increased synaptic efficacy of neurons in the spinal pain pathways) (Ji et al., 2003; Latremoliere and Woolf, 2009). Clinical recommended treatments include certain antidepressants, calcium channel α2-δ ligands, topical lidocaine, opioid analgesics, and antiepileptic medications depending on the patients’ symptoms and the degree of pain severity (Dworkin et al., 2007). Once neuropathic pain has been established it is very difficult to control. Even with well-established medications, effectiveness is unpredictable, dosing can be complicated, analgesic onset is delayed, and side effects are common (Dworkin et al., 2007). As a result it is necessary to investigate and develop more effective therapeutic approaches based on the pathophysiological mechanisms underlying the development of neuropathic pain.

While neuronal dysfunction is thought to be a primary important cause of pain, recent evidence suggests that alterations in glial cells including astrocytes and microglial cells play a critical role in the initiation of persistent pain (Watkins et al., 2001; Tan et al., 2009). Under pathophysiological conditions, glial cells release various pro-inflammatory cytokines, which bind to the receptors located on other glia and neurons resulting in neuronal excitation in the spinal cord dorsal horn (Chen et al., 2014; Choi et al., 2018). It has been suggested that the pro-inflammatory cytokine,IL-1β is rapidly increased and released from activated microglial cells during the early phases of peripheral nerve damage or inflammation (Samad et al., 2001; Fu et al., 2006; Choi et al., 2015). Furthermore, IL-1β modulates the function of other adjacent cells including neurons and thus serves as a key mediator in the interaction between glia and neurons in a variety of pain states (Kim et al., 2004; Ren and Dubner, 2008). While previous studies have suggested a possible relationship between IL-1β and nociceptive signal transmission, the detailed mechanisms involved in this process remain unclear.

Diverse neurosteroidogenic enzymes are expressed in brain and spinal cord and catalyze the local synthesis of neurosteroids in the central nervous system (Baulieu, 1998; Patte-Mensah et al., 2006). Among neurosteroidogenic enzymes, P450c17 catalyzes the conversion of PREG into DHEA (Compagnone and Mellon, 2000). DHEA and its sulfate ester DHEA-S are known as pronociceptive mediators in the nervous system (Kibaly et al., 2008; Yoon et al., 2010). In a previous study, we demonstrated that CCI of sciatic nerve increases both the protein and mRNA levels of P450c17 in spinal astrocytes during the early phase of neuropathic pain (Choi et al., 2019). Furthermore, intrathecal administration of the P450c17 inhibitor, ketoconazole during the induction phase of neuropathic pain resulted in a significant analgesic effect on the development of neuropathic pain evoked by sciatic nerve injury in mice. Since spinal P450c17 plays a critical role in the development of neuropathic pain, it is important to investigate the regulatory mechanisms by which astrocyte P450c17 expression and activation are increased following peripheral nerve injury in order to better understand the pathophysiological mechanisms underlying the early phase of neuropathic pain. Our recent studies have demonstrated that microglial IL-1β suppresses both the astrocyte-specific gap junction protein, connexin-43 expression and astrocyte activation during the early phase of carrageenan-induced inflammation ultimately inhibiting the development of contralateral MA (Choi et al., 2015, 2017). Thus, we hypothesize that during the early phase of neuropathic pain development spinal IL-1β inhibits astrocyte activation through modulation of astrocyte P450c17 expression.

Thus, the present study was designed to determine whether IL-1β modulates astrocyte P450c17 expression and astrocyte activation in the spinal cord and to determine whether this modulation alters the development of neuropathic pain following peripheral nerve injury. To accomplish this we investigated whether: (i) sciatic nerve injury increases the expression of IL-1β in the lumbar spinal cord dorsal horn; (ii) IL-1R1, a functional receptor of IL-1β, is expressed in spinal astrocytes; (iii) the blockade of IL-1R1 using the recombinant IL-1 receptor antagonist (IL-1ra) modulates astrocyte P450c17 expression and pathological astrocyte activation; and (iv) this modulation is associated with the development of MA induced by peripheral nerve injury.

## Materials and Methods

### Animals and Peripheral Nerve Injury Model

Four-week-old male Crl:CD1(ICR) mice (20–25 g) were purchased from the Laboratory Animal Center of Seoul National University (Seoul, South Korea). Animals had free access to food and water and were kept in temperature- and light-controlled rooms (23 ± 2°C, 12/12 h light/dark cycle) for at least 3 days prior to the beginning of the experiment. The experimental protocols for animal usage were reviewed and approved by the SNU Animal Care and Use Committee and were consistent with the Guide for the Care and Use of Laboratory Animals published by the United States National Institutes of Health (1985).

A CCI of the common sciatic nerve was performed according to the method described by Bennett and Xie with a minor modification (Bennett and Xie, 1988). Briefly, mice were anesthetized with 3% isoflurane in a mixture of N_2_O/O_2_ gas. The right sciatic nerve was exposed and three loose ligatures of 6-0 silk were placed around the nerve. Sham surgery was performed by exposing the sciatic nerve in the same manner, but without ligating the nerve. After surgery, animals recovered in clear plastic cages at 27°C with a thick layer of sawdust bedding.

### Drugs and Intrathecal Administration

The following drugs were used: recombinant interleukin-1 receptor antagonist (IL-1ra; 6, 20, and 60 ng); recombinant IL-1β (10 ng); Keto (1, 3, and 10 nmol), a P450c17 inhibitor; and FC (0.01, 0.03, and 0.1 nmol), an astrocyte metabolic inhibitor. IL-1ra and IL-1β were purchased from R&D Systems (Minneapolis, MN, United States), and ketoconazole and FC from Sigma–Aldrich (St. Louis, MO, United States). The doses of all drugs were selected based on doses previously used in the literature including our previous studies showing that these doses produce maximal effects with no detectable side effects (Souter et al., 2000; Choi et al., 2015, 2019). All drugs used for the behavioral experiments were administered intrathecally twice a day on postoperative days 0–3, which represents the induction phase of pain development. For the Western blot and immunohistochemical experiments drugs were administered once on postoperative day 0 and once on postoperative day 1. IL-1ra and IL-1β were dissolved in physiological saline, ketoconazole was dissolved in 5% DMSO in corn oil, and FC was dissolved in 0.05% 1N HCl in physiological saline. IL-1β was co-administrated with IL-1ra on days 0 and 1. Thus, a mixture of IL-1β and IL-1ra was injected immediately after the operation and again on day 1 post-surgery, then, the spinal cord was sampled on day 1. The intrathecal injection volume was 5 μl.

Intrathecal drug administration was performed using a 50 μl Hamilton syringe connected to a 30-gauge needle as previously described (Hylden and Wilcox, 1980). Mice were briefly anesthetized with 3% isoflurane in a mixture of N_2_O/O_2_ gas to prevent any handling-induced stress and to allow for a more accurate injection of drugs. The mouse was held tightly between the thumb and middle finger at the level of the both iliac crests, and the fifth lumbar spinous process was palpated with the index finger. The needle was inserted through the vertebral column into the L_5-6_ intervertebral space and successful insertion of the needle into the intrathecal space was determined by a tail flick response. Each drug was slowly injected over a 10 s period. Then, the needle was carefully removed from the intervertebral space. The drug control groups received an identical injection of vehicle.

### Assessment of Mechanical Allodynia

Pain behavioral testing was performed for ipsilateral (surgical-side) and contralateral hind paws of all animals 1 day before surgery in order to obtain normal baseline values of paw withdrawal responses to mechanical stimuli. Then, animals were randomly assigned to experimental and control groups. Animals were tested again at 1, 2, 3, 6, 9, and 14 days following CCI or sham surgery.

To assess nociceptive responses to innocuous mechanical stimuli (MA), we measured paw withdrawal response frequency (PWF) by using a von Frey filament with a force of 0.16 g (North Coast Medical, Morgan Hill, CA, United States) as described in a previous study from our laboratories (Moon et al., 2014). Mice were placed in acrylic cylinders on a wire mesh floor and allowed to habituate before testing. A von Frey filament was applied to the plantar surface of each hind paw for a 3 s period before being removed and we subsequently recorded whether there was a withdrawal of the hind limb to the filament. The filament was applied 10 times to the hind paw with a 10 s interval between each application. Then, the number of paw withdrawal responses was counted and the results of mechanical behavioral testing in the hind paw were expressed as a percent withdrawal response frequency (PWF, %), which represented the percentage of paw withdrawals out of the maximum of 10.

### Western Blot Assay

Animals were deeply anesthetized with 3% isoflurane in a mixture of N_2_O/O_2_ gas. The Western blot assay was performed as described previously with minor modifications (Choi et al., 2013). Different groups of mice were euthanized at each of the five different time points used in this study (0, 1, 3, 7, and 14 days after CCI surgery) in order to examine the time-course changes in IL-1β- and IL-1R1-expression. Another set of animals were euthanized at day 1 post-CCI surgery in order to determine the effect of IL-1ra treatment on GFAP- and P450c17-expression. Animals were perfused transcardially with calcium-free Tyrode’s solution and then the spinal cord was collected into an ice-cooled, saline-filled glass dish. The spinal cord dorsal horns of the lumbar enlargement segment were homogenized in lysis buffer (20 mM Tris–Hcl, 10 mM EGTA, 2 mM EDTA, pH 7.4 and proteinase inhibitors) containing 1% Triton X-100. Homogenates were subsequently centrifuged at 15,000 rpm for 40 min at 4°C and, then, the supernatant was used for Western blot analysis. The protein concentration was estimated by the Bradford dye assay (Bio-Rad Laboratories). Spinal cord homogenates (20–25 μg protein) were separated using 10% SDS-polyacrylamide gel electrophoresis and transferred to nitrocellulose membrane. After the blots had been washed with TBST (10 mM Tris–Hcl, pH 7.6, 150 mM NaCl and 0.05% Tween-20), the membranes were blocked with 5% skimmed milk for 1 h at RT and incubated at 4°C overnight with a primary antibody specific for IL-1β (rabbit polyclonal anti-IL1 beta antibody, 1:2,000, cat# ab9787, Abcam plc.), IL-1R1 (goat polyclonal anti-IL-1R1 antibody, 1:2,000, cat# AF771, R&D Systems), GFAP (mouse monoclonal anti-GFAP antibody, 1:2,000, cat# MAB360, Millipore Co.), P450c17 (rabbit monoclonal anti-cytochrome P450 17A1 antibody, 1:1,000, cat# ab125022, Abcam plc.), or β-actin (mouse monoclonal anti-β-actin antibody, 1:5,000, cat# sc-47778, Santa Cruz Biotechnology Inc.). After washing with TBST, membranes were incubated for 4 h at 4°C with horseradish peroxidase (HRP)-conjugated anti-rabbit, anti-goat, or anti-mouse antibody (1:10,000, Santa Cruz Biotechnology Inc.). The bands were visualized with enhanced chemiluminescence (Amersham Biosciences). The positive pixel area of specific bands was measured with a computer-assisted image analysis system and normalized against the corresponding β-actin loading control bands. The mean value of control groups was set at 100% and, then, the % change relative to the mean value of control groups was calculated for each group.

### Immunohistochemistry

Animals were deeply anesthetized with 3% isoflurane in a mixture of N_2_O/O_2_ gas and different groups of mice were euthanized at day 1 post-CCI surgery. The immunohistochemistry was performed as described previously with minor modifications (Roh et al., 2011). Mice were perfused transcardially with calcium-free Tyrode’s solution and subsequently with fixative containing 4% paraformaldehyde in 0.1 M phosphate buffer (pH 7.4). The spinal cords were collected after perfusion, post-fixed in the identical fixative for 2 h at RT and then placed in 30% sucrose in PBS (pH 7.4) at 4°C. Serial transverse sections (40 μm) of the L_4-5_ spinal cord were cut using a cryostat (Leica CM1520, Leica Biosystems, Germany). Transverse spinal cord sections were incubated in blocking solution for 1 h at RT and then incubated for 2 days at 4°C with a primary antibody specific for IL-1β (rabbit polyclonal anti-IL1 beta antibody, 1:1,000, cat# ab9787, Abcam plc.), IL-1R1 (goat polyclonal anti-IL-1R1 antibody, 1:100, cat# AF771, R&D Systems), P450c17 (rabbit monoclonal anti-cytochrome P450 17A1 antibody, 1:1,000, cat# ab125022, Abcam plc.), GFAP (mouse monoclonal anti-GFAP antibody, 1:1,000, cat# MAB360, Millipore Co.), NeuN (mouse monoclonal anti-NeuN antibody, 1:1,000, cat# MAB377, Millipore Co.), or Iba-1 (rabbit anti-Iba1 antibody, 1:500, cat# 019-19741, Wako Pure Chemical Industries, Ltd.). The primary antibodies were detected by incubating the tissue in Alexa Fluor^®^488 goat anti-mouse antibody (1:400, Life Technologies), Alexa Fluor^®^488 donkey anti-goat antibody (1:400, Life Technologies), Alexa Fluor^®^568 donkey anti-rabbit antibody (1:400, Life Technologies), or Alexa Fluor^®^555 donkey anti-mouse antibody (1:400, Life Technologies) for 90 min at RT. Tissue sections were mounted on slides and visualized with a confocal laser scanning microscope (Fluoview 300, Olympus, Tokyo, Japan; Nikon Eclipse TE2000-E, Nikon, Japan).

### Image Analysis

To analyze IL-1β- or GFAP-immunoreactive images, three to five spinal cord sections from the lumbar spinal cord segments were randomly selected from each animal, and were analyzed using a computer-assisted image analysis system (Metamorph version 7.7.2.0; Molecular Devices Corporation, PA, United States) as described in a previous study (Choi et al., 2016). To maintain a constant threshold for each image and to compensate for subtle variability of the immunostaining, we only measured areas that were 80% brightness in the range of intensity levels after background subtraction was performed. The positive pixel area of immunoreactive cells was quantified in the following three dorsal horn regions: (1) the SDH (laminae I and II); (2) the NP (laminae III and IV); and (3) the neck region (NECK, laminae V and VI). Then, the % threshold area [(positive pixel area/pixel area in each region) × 100] was counted. The average of % threshold area of immunoreactivity in each region per section from each animal was obtained and these values were averaged across each group and presented as group data. In addition, an average of 10 astrocytes from each animal with clear cell bodies and processes in lumbar spinal cord dorsal born were chosen for quantification of astrocyte morphology as described in a previous study (Lee et al., 2013). The astrocytes were analyzed with Metamorph software, generating data of cell body area (μm^2^) and length of processes (μm) from the soma.

For analysis of P450c17 colocalization in specific cell types in the spinal cord dorsal horn, pairs of fluorescent images were acquired on the confocal microscope as red and green channels as described in previous studies (Moon et al., 2014; Choi et al., 2019). Overlap of a pair of images was visualized in merged images as yellow pixels, which were considered colocalized. To analyze the extent of colocalization of P450c17 with GFAP (astrocytes) or NeuN (neurons), we directly quantified the number of cells showing cell type-specific nuclei that contained P450c17 immunolabelling. We quantified immunostaining in the following three dorsal horn regions: (1) the SDH (laminae I and II); (2) the NP (laminae III and IV); and (3) the NECK (laminae V and VI). All analytical procedures described above were performed blindly without knowledge of the experimental conditions.

### Data Presentation and Statistical Analysis

Data are expressed as the mean ± standard error of the mean (SEM). Statistical analyses were performed using Prism 5.0 (Graph Pad Software, San Diego, CA, United States). Repeated measures two-way (group and time) ANOVA was performed to determine differences in the behavioral data and One-way ANOVA was used to determine differences in the data obtained from Western blot assay and immunohistochemistry. *Post hoc* analysis was performed using a Bonferroni’s multiple comparison test to determine the *P*-value among experimental groups. Comparisons between 2 groups were analyzed by the two-tailed Student’s *t*-test. The level of significance was set at *P* < 0.05.

## Results

### Time Course Changes in IL-1β Expression in the Lumbar Spinal Cord Dorsal Horn of CCI Mice

**FIGURE 1:**
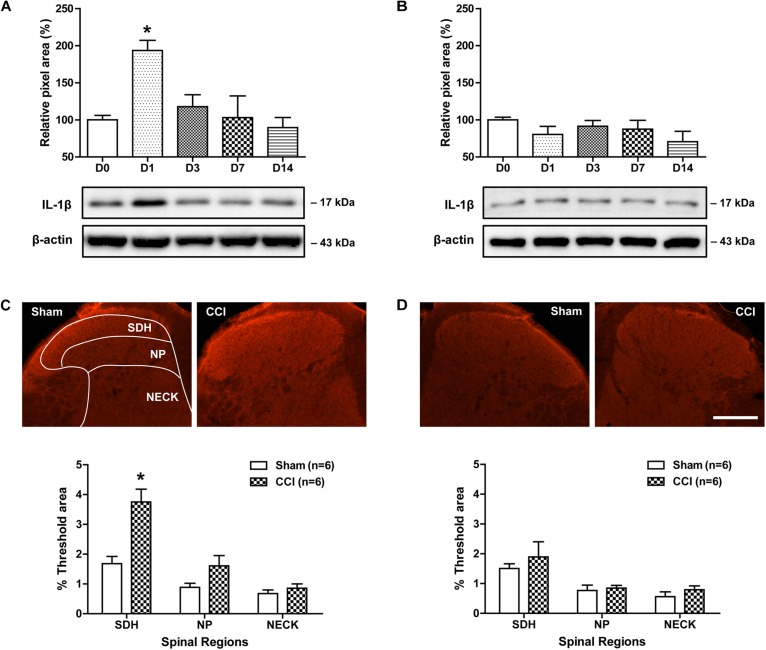
Time course of changes in the expression of IL-1β in the lumbar spinal cord dorsal horn of CCI mice. **(A,B)** The graphs depicting the changes in the protein expression of IL-1β are shown in the upper portion, while representative immunoblots are presented in the lower portion. Results of Western blot analysis showed that the protein expression of IL-1β significantly increased at day 1 post-CCI surgery in the ipsilateral lumbar spinal cord dorsal horn **(A)**, while the expression of IL-1β in the contralateral dorsal horn **(B)** did not change following CCI. The spinal cord dorsal horn was sampled at 0, 1, 3, 7, and 14 days after surgery. *n* = 6 mice/group. ^∗^*P* < 0.05 vs. D0. **(C,D)** Representative images showing the changes in IL-1β-immunoreactivity at postoperative day 1 in the lumbar spinal cord dorsal horn of CCI mice using immunohistochemistry. Fluorescent images of IL-1β in the superficial dorsal horn (SDH, lamina I-II), nucleus proprius (NP, lamina III-IV) and neck region (NECK, lamina V-VI) of sham and CCI mice. Fluorescence in the dorsal horn was quantitated using an image analysis system. Sciatic nerve injury significantly increased IL-1β-immunoreactivity in the SDH region of the ipsilateral lumbar spinal cord dorsal horn **(C)**, while IL-1β-immunoreactivity in the contralateral dorsal horn **(D)** did not change following CCI. *n* = 6 mice/group. ^∗^*P* < 0.05 vs. Sham. Scale bar = 100 μm.

To determine whether CCI of the sciatic nerve induces a significant change in spinal IL-1β expression, we examined changes in the protein expression of IL-1β over time after CCI using a Western blot analysis. Sciatic nerve injury significantly increased the amount of IL-1β protein in the ipsilateral lumbar spinal cord dorsal horn at day 1 post-surgery as compared with control mice. Subsequently IL-1β expression was dramatically decreased from 3 days to 14 post-surgery [Figure 1A; *F*(4,25) = 5.728, *P* = 0.0021]. By contrast, the amount of IL-1β in the contralateral lumbar spinal cord dorsal horn did not change following sciatic nerve injury as compared with the control group [Figure 1B; *F*(4,25) = 1.175, *P* = 0.3611]. We also examined changes in IL-1β-immunoreactivity in the lumbar spinal cord dorsal horn at day 1 post-surgery using an immunohistochemical approach. Sciatic nerve injury significantly increased IL-1β-immunoreactivity in the SDH (laminae I-II) region of the ipsilateral lumbar spinal cord dorsal horn as compared to that of the sham surgery animals [Figure 1C; SDH: *t*(10) = 4.202, *P* = 0.0018; NP: *t*(10) = 1.936, *P* = 0.0816; NECK: *t*(10) = 0.9577, *P* = 0.3608]. By contrast, IL-1β-immunoreactivity in the contralateral lumbar spinal cord dorsal horn did not change following sciatic nerve injury as compared with the control group [Figure 1D; SDH: *t*(10) = 0.7205, *P* = 0.4877; NP: *t*(10) = 0.4023, *P* = 0.6959; NECK: *t*(10) = 1.112, *P* = 0.2921]. These results show that sciatic nerve injury induces an early transient increase in IL-1β expression, especially in the SDH (laminae I-II) of the ipsilateral lumbar spinal cord.

### Time Course Changes in Spinal IL-1R1 Expression and Effects of IL-1ra on the Development of Mechanical Allodynia in CCI Mice

**FIGURE 2:**
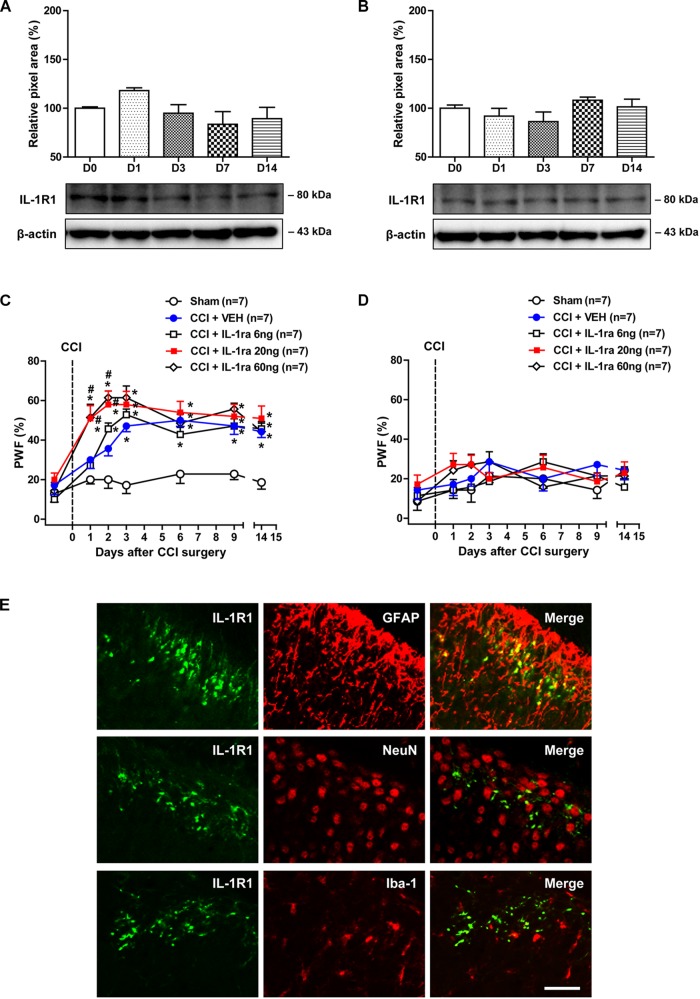
Time course of changes in the expression of IL-1R1 and the effects of administration of an IL-1 receptor antagonist (IL-1ra; 6, 20, or 60 ng) on the CCI-induced development of MA. **(A,B)** The graphs depicting the changes in the protein expression of IL-1R1 are shown in the upper portion, while representative immunoblots are presented in the lower portion. Results of Western blot analysis showed that the protein expression of IL-1R1 did not change in the ipsilateral **(A)** and contralateral **(B)** lumbar spinal cord dorsal horn following CCI. The spinal cord dorsal horn was sampled at 0, 1, 3, 7, and 14 days after surgery. *n* = 6 mice/group. **(C,D)** Paw withdrawal frequency (PWF, %) was measured in hind paws using a von-Frey filament (0.16 g). Sciatic nerve injury increased the PWF to mechanical stimuli in the ipsilateral hind paw from day 3 post-surgery, while administration of IL-1ra (20 or 60 ng) induced a significant increase in the PWF beginning on day 1 post-surgery **(C)**. On the other hand, administration of IL-1ra had no effect on the PWF in the contralateral hind paw **(D)**. *n* = 7 mice/group. ^∗^*P* < 0.05 vs. Sham; #*P* < 0.05 vs. vehicle-treated group. **(E)** Using a double immunohistochemical approach these representative images depict colocalization (yellow) of IL-1R1 (green) with GFAP (red, a marker of astrocytes), but not with NeuN (red, a marker of neurons) or Iba-1 (red, a marker of microglial cells) at day 1 post-surgery in the superficial dorsal horn of lumbar spinal cord in CCI mice. Scale bar = 40 μm.

Involvement of interleukin-1β exerts its effects by acting on the membrane bound IL-1R1, which activates signal transduction upon IL-1β stimulation (Sims et al., 1993). Thus in the next part of our study, we examined changes in the protein expression of IL-1R1 over time following CCI using a Western blot analysis. The protein amount of IL-1R1 in the ipsilateral [Figure 2A; *F*(4,25) = 2.243, *P* = 0.0930] and contralateral [Figure 2B; *F*(4,25) = 1.484, *P* = 0.2369] lumbar spinal cord dorsal horn did not change following sciatic nerve injury as compared with the control group.

In order to determine if the CCI-induced increase in the expression of IL-1β at day 1 post-surgery modulates the development of MA, we examined the effect of administration of the IL-1 receptor antagonist (IL-1ra; 6, 20, or 60 ng) on the development of MA. CCI of the sciatic nerve increased the PWF (%) to innocuous mechanical stimuli (MA) in the ipsilateral hind paw from 3 days post-CCI surgery as compared with sham group (Figure 2C). IL-1ra was administered intrathecally during the induction phase of neuropathic pain (from days 0 to 3 post-surgery) in order to neutralize spinal IL-1β signaling. Intrathecal administration of IL-1ra (20 or 60 ng) induced an early increase in the ipsilateral PWF beginning at day 1 post-surgery [Figure 2C; Group: *F*(4,210) = 42.59, *P* < 0.0001; Time: *F*(6,210) = 25.98, *P* < 0.0001; Interaction: *F*(24,210) = 1.928, *P* = 0.0074]. By contrast, i.t. administration of IL-1ra (6, 20, or 60 ng) during the induction phase of neuropathic pain (from days 0 to 3 post-surgery) had no effect on the PWF in the contralateral hind paw [Figure 2D; Group: *F*(4,210) = 1.908, *P* = 0.1104; Time: *F*(6,210) = 2.987, *P* = 0.0081; Interaction: *F*(24,210) = 0.8927, *P* = 0.6120].

Double immunostaining was performed with antibodies against the IL-1R1 and several cell specific markers to determine the cellular localization of IL-1R1 expression in the lumbar spinal cord dorsal horn at day 1 post-surgery (this day was selected because IL-1β expression was significantly increased at this timepoint). IL-1R1 expression was colocalized in GFAP-positive astrocytes in the SDHs, whereas there was no colocalization of IL-1R1 in NeuN-positive neurons or Iba-1-positive microglial cells in the SDHs of lumbar spinal cord (Figure 2E). These results demonstrate that the early activation of astrocyte IL-1R1 induced by increased IL-1β controls the development of MA following sciatic nerve injury.

### Effects of IL-1ra on the Pathophysiological Changes in Astrocyte Activation in the Lumbar Spinal Cord Dorsal Horn of CCI Mice

**FIGURE 3:**
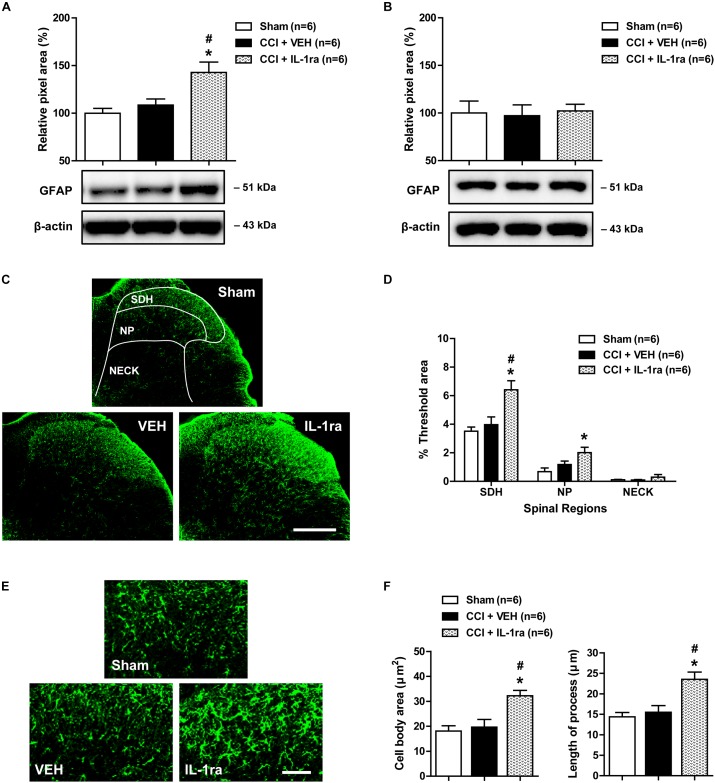
Effects of IL-1 receptor antagonist (IL-1ra, 20 ng) administration on the expression of GFAP in the lumbar spinal cord dorsal horn of CCI mice. **(A)** Results of Western blot analysis showing that the protein expression of GFAP did not change following CCI at day 1 post-CCI surgery, while administration of IL-1ra increased the expression of GFAP in the ipsilateral lumbar spinal cord dorsal horn. **(B)** The expression of GFAP in the contralateral dorsal horn did not show any change after CCI and IL-1ra administration. *n* = 6 mice/group. **(C)** Representative images showing the changes in GFAP expression at day 1 post-surgery in the ipsilateral lumbar spinal cord dorsal horn of CCI mice using immunohistochemistry. Scale bar = 200 μm. **(D)** The immunofluorescence of GFAP in the superficial dorsal horn (SDH, lamina I-II), nucleus proprius (NP, lamina III-IV) and neck region (NECK, lamina V-VI) of mice was quantitated using an image analysis system. GFAP-immunoreactivity did not change following CCI at day 1 post-CCI surgery, while the administration of IL-1ra increased the expression of GFAP in the ipsilateral lumbar spinal cord dorsal horn. **(E)** Higher magnification images are shown in panel **(E)**. Scale bar = 40 μm. **(F)** The cell body area (μm^2^) and the length of processes (μm) radiating from the soma of astrocytes did not change following CCI at day 1 post-surgery, while administration of IL-1ra increased the cell body area (μm^2^) and the length of processes (μm). The spinal cord dorsal horn was sampled at day 1 post-surgery. *n* = 6 mice/group. ^∗^*P* < 0.05 vs. Sham; #*P* < 0.05 vs. vehicle-treated group.

To determine whether the CCI-induced early increase in spinal IL-1β expression modulates pathophysiological changes in astrocyte activation, we examined the effect of IL-1ra on the expression of GFAP, a major protein constituent of glial filaments in astrocytes of the central nervous system (Eng, 1985), in the lumbar spinal cord dorsal horn of CCI mice using Western blot analysis and immunohistochemistry. Results of Western blot analysis revealed that there was no change in GFAP expression in the ipsilateral lumbar spinal cord dorsal horn at day 1 post-CCI surgery as compared with sham group (Figure 3A). By contrast, intrathecal administration of IL-1ra (20 ng) on days 0 and 1 post-surgery significantly increased the expression of GFAP at day 1 post-surgery as compared with sham and vehicle-treated groups [Figure 3A; *F*(2,15) = 8.306, *P* = 0.0037]. However, there was no change in the expression of GFAP in the contralateral spinal cord dorsal horn after CCI and IL-1ra (20 ng) administration [Figure 3B; *F*(2,15) = 0.05556, *P* = 0.9461]. Immunohistochemical analysis showed that GFAP-immunoreactivity did not change in the ipsilateral lumbar spinal cord dorsal horn of CCI mice at day 1 post-surgery as compared with the sham group (Figure 3C,D). Intrathecal administration of IL-1ra significantly increased GFAP-immunoreactivity in the SDH (laminae I-II) and NP (laminae III-IV) regions at day 1 post-surgery as compared with sham group [Figure 3C,D; SDH: *F*(2,15) = 9.280, *P* = 0.0024; NP: *F*(2,15) = 4.970, *P* = 0.0221; NECK: *F*(2,15) = 1.011, *P* = 0.3875] further supporting what was observed in our Western blot analysis. When viewed under higher magnification astrocytes in the ipsilateral lumbar dorsal horn show hypertrophy with enlarged cell bodies [*F*(2,15) = 9.639, *P* = 0.0020] and elongated processes radiating from the soma [*F*(2,15) = 10.91, *P* = 0.012] in the IL-1ra-treated group at day 1 post-surgery as compared with sham and vehicle-treated groups (Figure 3E,F). These results suggest that the early activation of astrocyte IL-1R1 controls the induction of the pathophysiological changes in spinal astrocyte activation associated with CCI.

### Effects of IL-1ra on the Expression of Astrocyte P450c17 in the Lumbar Spinal Cord Dorsal Horn of CCI Mice

**FIGURE 4:**
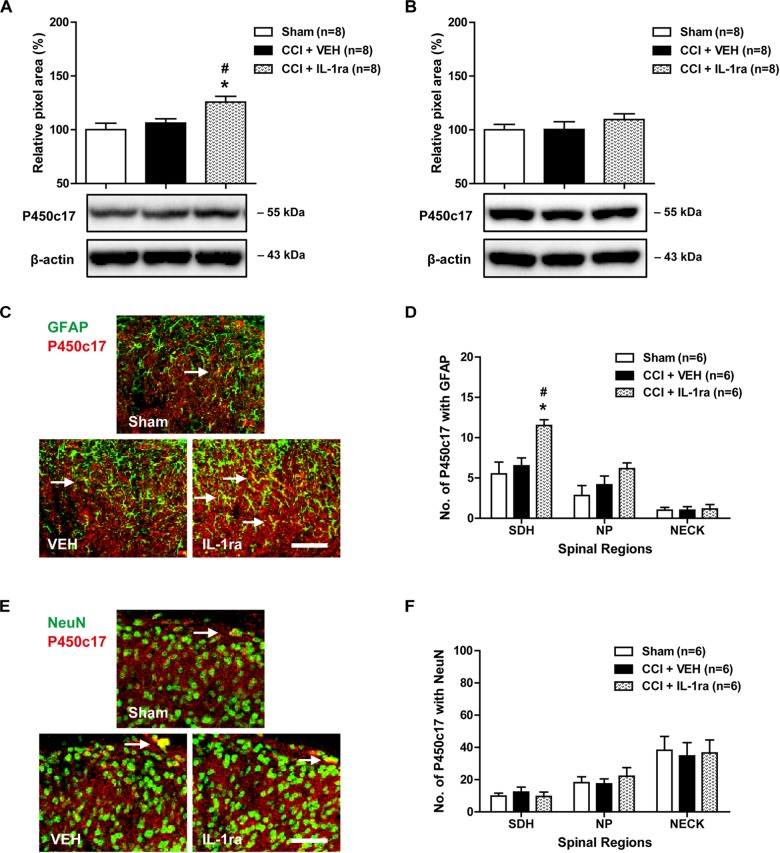
Effects of IL-1 receptor antagonist (IL-1ra, 20 ng) administration on the expression of P450c17 in the lumbar spinal cord dorsal horn of CCI mice. **(A)** Results of Western blot analysis showed that the protein expression of P450c17 did not change at day 1 post-CCI surgery, while administration of IL-1ra increased the protein expression of P450c17 in the ipsilateral spinal cord dorsal horn. **(B)** The expression of P450c17 in the contralateral dorsal horn did not show any change after CCI and IL-1ra administration. *n* = 8 mice/group. **(C–F)** Using a double immunohistochemical approach these representative images depict colocalization (yellow) of P450c17 with GFAP (**C**; green, a marker of astrocytes) or NeuN (E; green, a marker of neurons) at day 1 post-surgery in the ipsilateral lumbar spinal cord dorsal horn of CCI mice. Scale bar = 50 μm. Colocalization was quantitated in the superficial dorsal horn (SDH, lamina I-II), nucleus proprius (NP, lamina III-IV) and neck region (NECK, lamina V-VI). The number of P450c17-immunostained astrocytes **(D)** did not change following CCI at day 1 post-CCI surgery, while administration of IL-1ra increased the number of P450c17-immunostained astrocytes in the SDH region of the ipsilateral lumbar spinal cord. The number of P450c17-immunostained neurons **(F)** did not change following CCI and treatment with IL-1ra. The spinal cord dorsal horn was sampled at day 1 post-surgery. *n* = 6 mice/group. ^∗^*P* < 0.05 vs. Sham; #*P* < 0.05 vs. vehicle-treated group.

To determine whether the CCI-induced early increase in spinal IL-1β expression modulates the protein expression of P450c17, we examined the effect of IL-1ra on the P450c17 expression in the lumbar spinal cord dorsal horn using Western blot analysis and immunohistochemistry. There was no change in the P450c17 expression in the ipsilateral lumbar spinal cord dorsal horn at day 1 post-CCI surgery as compared with the sham group. Conversely intrathecal administration of IL-1ra (20 ng) on days 0 and 1 post-surgery significantly increased the expression of P450c17 at day 1 post-surgery as compared with sham and vehicle-treated groups [Figure 4A; *F*(2,21) = 6.507, *P* = 0.0063]. However, there was no change in the expression of P450c17 in the contralateral spinal cord dorsal horn after CCI and IL-1ra (20 ng) administration [Figure 4B; *F*(2,21) = 0.8167, *P* = 0.4554]. In addition, the number of P450c17-immunostained astrocytes did not change in the ipsilateral lumbar spinal cord dorsal horn at day 1 post-surgery as compared with sham group (Figure 4C,D). Administration of IL-1ra significantly increased the number of P450c17-immunostained astrocytes in the SDH (laminae I-II) region at day 1 post-surgery as compared with the sham and vehicle-treated groups [Figure 4C,D; SDH: *F*(2,15) = 8.416, *P* = 0.0035; NP: *F*(2,15) = 2.681, *P* = 0.1011; NECK: *F*(2,15) = 0.04425, *P* = 0.9568]. On the other hand, there was no change in the number of P450c17-immunostained neurons in the ipsilateral lumbar spinal cord dorsal horn at day 1 post-surgery and no significant difference was observed between the vehicle-treated and IL-1ra-treated groups for P450c17 immunostaining in neurons [Figure 4E,F; SDH: *F*(2,15) = 0.3540, *P* = 0.7076; NP: *F*(2,15) = 0.3894, *P* = 0.6841; NECK: *F*(2,15) = 0.04407, *P* = 0.9570]. These results suggest that the early activation of astrocyte IL-1R1 controls the expression of P450c17, which is increased in spinal astrocytes following CCI.

### Effects of IL-1β on the IL-1ra-Induced Early Increase in Spinal P450c17 Expression and Astrocyte Activation in CCI Mice

**FIGURE 5:**
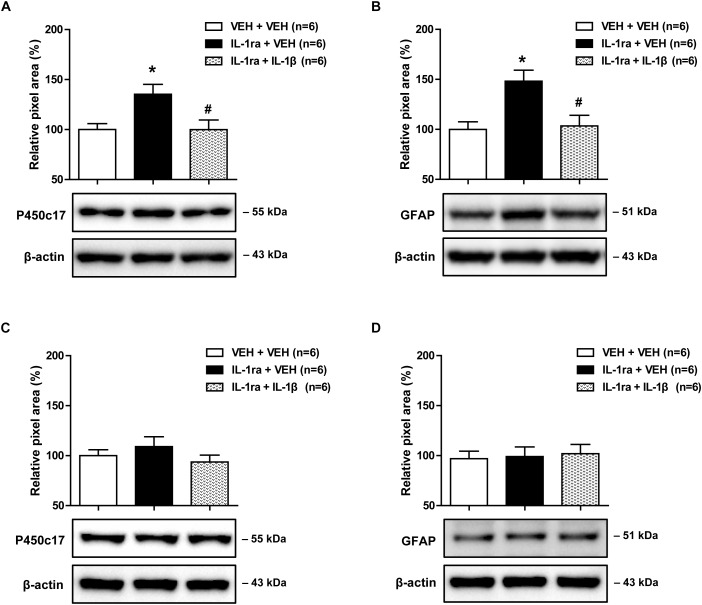
Effects of IL-1β (10 ng) on the early increases in the expression of spinal P450c17 and astrocyte activation in IL-1 receptor antagonist (IL-1ra, 20 ng) treated CCI mice. **(A)** Results of Western blot analysis showed that the protein expression of P450c17 in the ipsilateral spinal cord dorsal horn was increased at day 1 post-CCI surgery by administration of IL-1ra, and this increase was blocked by the co-administration of IL-1β (10 ng) with IL-1ra. **(B)** Results of Western blot analysis showed that the protein expression of GFAP in the ipsilateral spinal cord dorsal horn was increased at day 1 post-CCI surgery by administration of IL-1ra, and this increase was blocked by co-administration of IL-1β (10 ng) with IL-1ra. **(C,D)** The expression of P450c17 **(C)** and GFAP **(D)** in the contralateral dorsal horn did not show any change after drug administration. The spinal cord dorsal horn was sampled at day 1 post-surgery. *n* = 6 mice/group. ^∗^*P* < 0.05 vs. vehicle-treated group; #*P* < 0.05 vs. IL-1ra-treated group.

Next, we used Western blot analysis to examine whether blocking the direct effect of IL-1ra with spinal IL-1β would alter the increase in astrocyte P450c17 expression and astrocyte activation in the lumbar spinal cord dorsal horn. Intrathecal administration of IL-1ra (20 ng) on days 0 and 1 post-surgery significantly increased the expression of P450c17 in the ipsilateral spinal cord dorsal horn at day 1 post-surgery as compared with the vehicle-treated groups. This increase in P450c17 expression was significantly reduced by the intrathecal co-administration with IL-1β (10 ng in combination with IL-1ra) [Figure 5A; *F*(2,15) = 5.556, *P* = 0.0156]. In addition, intrathecal administration of IL-1ra (20 ng) on days 0 and 1 post-surgery significantly increased the expression of GFAP in the ipsilateral spinal cord dorsal horn at the 1 day post-surgery timepoint as compared with the vehicle-treated groups, and this increase was significantly reduced by the intrathecal administration of IL-1ra with IL-1β (10 ng in combination with IL-1ra) [Figure 5B; *F*(2,15) = 7.399, *P* = 0.0058]. However, there was no change in the expression of P450c17 [Figure 5C; *F*(2,15) = 1.010, *P* = 0.3876] and GFAP [Figure 5D; *F*(2,15) = 0.07990, *P* = 0.9236] in the contralateral spinal cord dorsal horn after drug administration. These results show that i.t. administration of IL-1β blocks the early increase in spinal P450c17 expression and astrocyte activation induced by blockade of spinal IL-1 receptors in CCI mice.

### Effects of Ketoconazole or Fluorocitrate on the IL-1ra-Induced Early Development of Mechanical Allodynia in CCI Mice

**FIGURE 6:**
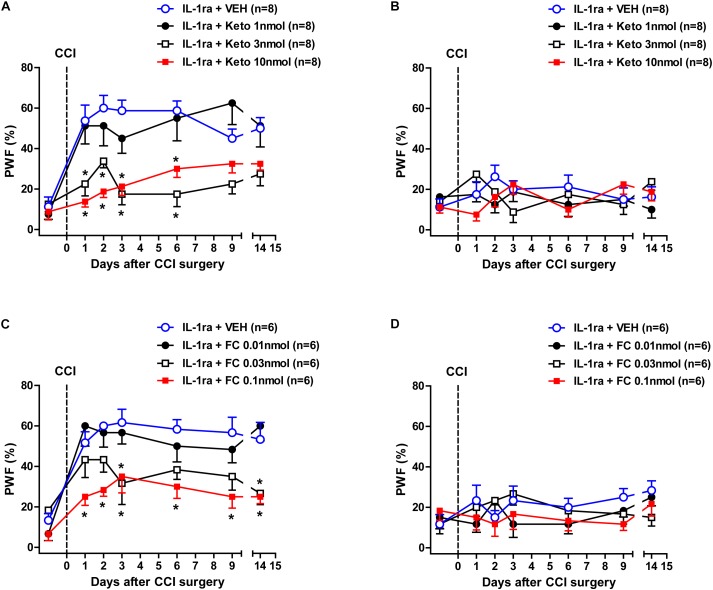
Effects of the P450c17 inhibitor, ketoconazole (Keto, 1, 3, and 10 nmol) or the astrocyte metabolic inhibitor, fluorocitrate (FC, 0.01, 0.03, and 0.1 nmol) on the early development of mechanical allodynia in IL-1 receptor antagonist (IL-1ra, 20 ng) treated CCI mice. **(A–D)** Paw withdrawal frequency (PWF, %) was measured in hind paws using a von-Frey filament (0.16 g). IL-1ra administration induced an early increase in the PWF of the ipsilateral hind paw and this was significantly suppressed by the co-administration of ketoconazole with IL-1ra **(A)**. On the other hand, administration of ketoconazole in association with IL-1ra had no effect on the PWF in the contralateral hind paw **(B)**. *n* = 8 mice/group. ^∗^*P* < 0.05 vs. IL-1ra-treated group. In addition, IL-1ra-induced an early increase in the PWF of the ipsilateral hind paw was significantly suppressed by the co-administration of fluorocitrate with IL-1ra **(C)**. Conversely, administration of fluorocitrate in association with IL-1ra had no effect on the PWF of the contralateral hind paw **(D)**. *n* = 6 mice/group. ^∗^*P* < 0.05 vs. IL-1ra-treated group.

We next examined whether intrathecal administration of the P450c17 inhibitor, ketoconazole or the astrocyte metabolic inhibitor, FC, would change the early development of MA induced by IL-1ra administration. Intrathecal administration of IL-1ra (20 ng) from days 0 to 3 post-surgery increased the PWF (%) to innocuous mechanical stimuli (MA) in the ipsilateral hind paw beginning at 1 day post-CCI surgery as compared with normal baseline values (Figure 6A). Treatment with the P450c17 inhibitor, ketoconazole (1, 3, or 10 nmol together with IL-1ra), significantly reduced this CCI/IL-1ra-induced development of MA in the ipsilateral hind paw [Figure 6A; Group: *F*(3,196) = 37.58, *P* < 0.0001; Time: *F*(6,196) = 12.81, *P* < 0.0001; Interaction: *F*(18,196) = 2.189, *P* = 0.0047]. By contrast, i.t. co-administration of ketoconazole with IL-1ra (20 ng) during the induction phase of neuropathic pain (from days 0 to 3 post-surgery) had no effect on the PWF in the contralateral hind paw [Figure 6B; Group: *F*(3,196) = 0.8633, *P* = 0.4611; Time: *F*(6,196) = 0.5662, *P* = 0.7570; Interaction: *F*(18,196) = 1.483, *P* = 0.0990]. In addition, intrathecal administration of the astrocyte metabolic inhibitor, FC (0.01, 0.03, or 0.1 nmol together with IL-1ra) significantly reduced the CCI-induced development of MA in the ipsilateral paw that developed beginning 1 day post-CCI surgery following IL-1ra administration [Figure 6C; Group: *F*(3,140) = 26.39, *P* < 0.0001; Time: *F*(6,140) = 16.20, *P* < 0.0001; Interaction: *F*(18,140) = 1.391, *P* = 0.1447]. By contrast, i.t. co-administration of FC with IL-1ra (20 ng) during the induction phase of neuropathic pain (from days 0 to 3 post-surgery) had no effect on the PWF in the contralateral hind paw [Figure 6D; Group: *F*(3,140) = 1.445, *P* = 0.2325; Time: *F*(6,140) = 1.002, *P* = 0.4268; Interaction: *F*(18,140) = 0.9128, *P* = 0.5643]. These results suggest that early increases in P450c17 expression and astrocyte activation contribute to the early development of ipsilateral MA induced by the inhibition of spinal IL-1 receptors in CCI mice.

## Discussion

This study demonstrates three important novel findings. First, CCI-induced sciatic nerve injury produces an early, but transient, increase in IL-1β expression in the SDH of the ipsilateral lumbar spinal cord. Moreover, we demonstrate that the IL-1 receptor type 1 (IL-1R1), a functional receptor of IL-1β, is expressed in GFAP-positive astrocytes, which are also exclusively located in the SDH region. Secondly, blockade of IL-1R1 with intrathecal administration of the IL-1 receptor antagonist (IL-1ra) during the early phase of peripheral neuropathy increased the expression of both astrocyte P450c17 and GFAP in the spinal cord dorsal horn, and this increase was blocked by IL-1β administration. Finally, intrathecal administration of IL-1ra significantly facilitated the development of MA, and this facilitation was suppressed by intrathecal administration of either the P450c17 inhibitor, ketoconazole or the astrocyte metabolic inhibitor, FC. Collectively, we believe that this data significantly increases our understanding of one of the mechanisms underlying the development of neuropathic pain by demonstrating that the modulation of astrocyte P450c17 expression is closely associated with an early increased release of IL-1β in the lumbar spinal cord dorsal horn and this P450c17 modulation of IL-1β ultimately affects the development of MA induced by peripheral nerve injury. In the present study, we demonstrated that IL-1β expression was significantly increased in the SDH (laminae I-II) region of the ipsilateral spinal cord at 1 day following peripheral nerve injury. These results suggest that the expression of IL-1β is transiently upregulated during the early phase of neuropathic pain, which in turn can influence the activity of various adjacent cells in the SDH region of the spinal cord. There are two receptors for the interleukin-1 (IL-1) have been characterized; IL-1 type 1 receptor (IL-1R1) and IL-1 type 2 receptor (IL-1R2) (Ren and Torres, 2009). IL-1R2 lacks an intracellular domain and is incapable of signal transduction, while IL-1R1 is a transmembrane molecule that is responsible for IL-1β signal transducing (Sims et al., 1993). In the present study, double immunofluorescence staining of the IL-1 receptor type 1 and GFAP, a major protein constituent of glial filaments in astrocytes of the central nervous system (Eng, 1985), strongly supports a close interaction of IL-1β signaling with spinal astrocytes. Furthermore, blockade of IL-1R1 with an IL-1 receptor antagonist during the induction phase of neuropathic pain facilitated astrocyte hypertrophy with enlarged cell bodies and elongated processes radiating from the soma especially in the SDH, where primary afferent nociceptive C-fibers and myelinated A-fibers terminate and synapse with second order neurons (Todd, 2010). These results strongly suggest the possibility that the early transient increase in IL-1β plays an important role in the control of the pathological changes in adjacent astrocytes via binding to the IL-1 type 1 receptors on astrocytes located in SDH region, ultimately affecting nociceptive synaptic transmission following peripheral nerve injury.

It has become increasingly evident that IL-1β is released from activated microglial cells in response to a variety of inflammatory stimuli (Clark et al., 2010; Choi et al., 2015). Paclitaxel triggers the elevation of Ca^2+^ levels in microglial cells by activation of microglial toll like receptor 4, which leads to the rapid release of IL-1β from spinal microglial cells (Yan et al., 2019). In addition, the microglial P2X7 receptor is involved in the lipopolysaccharide (LPS)-induced release of IL-1β in the spinal cord dorsal horn (Clark et al., 2010). While astrocytes are also able to produce IL-1β, astrocytes are less likely to respond directly to LPS, but rather respond to cytokines that are released from microglial cells activated by LPS (Lee et al., 1993). Moreover, higher levels of cytokines are induced in microglial cells than in astrocytes (Lee et al., 1993). Thus, we propose that spinal microglial cells are a major source of IL-1β during the early phase of peripheral neuropathy. Furthermore, it has been reported that IL-4, -10, and -13 play a role in producing an analgesic effect in experimental inflammatory pain models suggesting that the endogenous release of these cytokines may limit the development of the nociceptive response during inflammatory pain reactions (Vale et al., 2003). This antinociceptive effect seems to be mediated through a peripheral mechanism involving the inhibition of the release of proinflammatory cytokines (Vale et al., 2003). Since microglial cells express the mRNAs for both the IL-4 and IL-10 cytokine receptors and since these cytokines function as negative regulators of microglial functions (Sawada et al., 1999), it is important to do future investigations on the role of diverse cytokines in the central mechanisms underlying the development of antinociception in inflammatory pain conditions.

In the present study, early blockade of the IL-1 receptor with an IL-1 receptor antagonist significantly enhances the expression of P450c17 in astrocytes. In addition, inhibition of spinal P450c17 with the P450c17 inhibitor, ketoconazole blocks the early development of MA induced by administration of an IL-1 receptor antagonist, suggesting that the nerve injury-induced increase in IL-1β may produce an analgesic effect through inhibition of both the expression of astrocyte P450c17 and the concomitant production of neurosteroids in astrocytes as possible control mechanisms against the development of neuropathic pain. There are several studies supporting our concept that IL-1β has an analgesic effect on nociceptive signal transmission in the central nervous system. Souter et al. showed that intrathecal IL-1β dose-dependently suppressed carrageenan-induced inflammatory pain and this was not opioid-dependent (Souter et al., 2000). Kim et al. observed that IL-1β injected intracisternally produced antinociceptive effects in an NMDA-evoked pain model of the orofacial area, while the antinociceptive effect was mediated by an opioid pathway (Kim et al., 2004). Collectively these results suggest that IL-1β-induced analgesia occurs via different mechanisms in the spinal cord and at higher levels of the neuroaxis.

Interleukin-1 is a family of 11 cytokines that are the products of separate genes and play a central role in the regulation of immune and inflammatory responses (Rothwell and Luheshi, 2000). IL-1α and IL-1β are the agonists of IL-1 receptors, while IL-1ra binds to IL-1 receptors but does not induce any intracellular response (Arend et al., 1998). In the present study, IL-1ra induced an early increase in the expression of GFAP and P450c17, which was prevented by the co-administration of IL-1β with IL-1ra. Since not only IL-1β, but also endogenous IL-1α and IL-1ra are able to bind to IL-1 receptors, these results suggest that the effect of exogenous IL-1ra on both astrocyte activity and P450c17 expression is mediated in large part by blockade of IL-1β’s action in this neuropathic pain model. It has been suggested that IL-1α and IL-1β act on the same receptor to differentially influence nociceptive transmission and the neuropathic pain response (Mika et al., 2008). In addition, Andre et al. suggested that IL-1α and IL-1β are identical in their ability to initiate downstream activation of mitogen-activated protein kinases and nuclear factor-kappa B, but IL-1β caused IL-6 release more potent than IL-1α, suggesting the possible existence of additional signaling pathway activated by IL-1β (Andre et al., 2005). The detail mechanisms underlying the effects of IL-1β in the central nervous system remain unclear, but the presence of endogenous IL-1α and IL-1ra suggests the possibility that this IL-1 family may influence the action of IL-1β by competing with that for binding sites of the receptor.

In a previous study from our laboratories, we showed that the protein expression of GFAP and P450c17 in the ipsilateral lumbar spinal cord dorsal horn was statistically increased at 3 days post-CCI surgery and that inhibition of astrocytes or P450c17 during this induction phase (from days 0 to 3 post-surgery) had significant analgesic effects on the CCI-induced development of neuropathic pain (Moon et al., 2014; Choi et al., 2019). By contrast, the protein expression of GFAP and P450c17 was gradually decreased and was not statistically different in the spinal cord dorsal horn of day 14 post-surgery mice compared with control mice and inhibition of P450c17 during this maintenance phase (from days 14 to 17 post-surgery) had no effect on the neuropathic pain that had already developed (data not shown). These results raise the possibility that during the maintenance phase, the stimulatory effect of P450c17 disappears and/or another inhibitory control factor appears. This should be further investigated in order to understand the different mechanisms associated with the induction versus the maintenance phases of neuropathic pain.

In the present study, we focused our investigation on whether IL-1β can have a negative modulatory effect on the development of neuropathic pain and thus serve as a transient negative control mechanism. By contrast, IL-1β has been reported to induce hyperalgesia by exerting robust cellular inflammatory actions as a pro-inflammatory cytokine *in vivo*. These actions are supported by several studies showing that peripheral IL-1β contributes to the development of inflammatory pain hypersensitivity by increasing cyclooxygenase-2 expression, leading to the release of prostanoids, which sensitize peripheral nociceptors (Samad et al., 2001). It has also been reported that spinal IL-1β increased the phosphorylation of the NMDA receptor GluN1 subunit and facilitated pain in a rat model of inflammatory pain (Zhang et al., 2008). While several studies using animal models have demonstrated that the inhibition of IL-1 significantly inhibits persistent pain, this did not occur in our study. In this regard, Gabay et al. (2011) showed that chronic pharmacological blockade of IL-1 signaling markedly reduced neuropathic pain symptoms, as reflected by attenuated MA. Conversely our results indicate that blockade of IL-1 signaling produces a robust but temporary antinociceptive effect. This discrepancy may be due to: (1) the early timepoint examined in this study; (2) differences in the animal models being examined; (3) the different time course of nociception in each model; (4) different routes of administration of IL-1ra or other drugs; and/or (5) the nervous system location being examined. These different experimental conditions can alter the micro-environment of the nervous system by changing the expression patterns of the IL-1 receptor, the activation of adjacent glial cells, and/or the release of other cytokines. Thus, the effect of IL-1β can be modified based on changes to any of these parameters. Since the evidence suggests that IL-1β can have contradictory roles, analgesia versus hyperalgesia, on nociception, it is important that the experimental design of future studies take this into account in order to exploit specific targeting of the IL-1β pathway. In this regard we plan to investigate the mechanisms underlying cytokine-induced modulation of pain transmission in more detail in future studies.

## Conclusion

In conclusion, the present study demonstrates that the expression of IL-1β is significantly increased in the ipsilateral lumbar spinal cord dorsal horn of CCI mice and that blockade of central IL-1β signaling by intrathecal administration of IL-1 receptor antagonist during the induction phase of neuropathic pain not only facilitates the development of MA, but also increases both astrocyte P450c17 expression and pathological astrocyte activation in CCI mice. Collectively these results suggest that early increased spinal IL-1β plays an important role as a transient analgesic mechanism that controls the development of neuropathic pain by inhibiting both the expression of astrocyte P450c17 and the activation of spinal astrocytes following peripheral nerve injury. This study offers new insights into a potential, but transient analgesic role of IL-1β in nociceptive processing at the level of the spinal cord and suggests that development of interleukin-1 receptor agonists may serve as novel and selective therapeutic agents for the prevention of the development of neuropathic pain following peripheral nerve injury.

## Data Availability

The datasets for this manuscript are not publicly available because of a security issue. Requests to access the datasets should be directed to J-HL, jhl1101@snu.ac.kr.

## Ethics Statement

The experimental protocols for animal usage were reviewed and approved by the SNU Animal Care and Use Committee and were consistent with the Guide for the Care and Use of Laboratory Animals published by the United States National Institutes of Health (1985).

## Author Contributions

S-RC contributed to the design of the study, acquisition and analysis of data, and drafting the manuscript. H-JH assisted molecular biological techniques and the data analysis of molecular biological experiments. AB was involved with data analysis and revised the manuscript for important intellectual content. J-HL contributed to the conception of the study, interpretation of data, and final approval of the version to be submitted.

## Conflict of Interest Statement

The authors declare that the research was conducted in the absence of any commercial or financial relationships that could be construed as a potential conflict of interest.

## Abbreviations

CCI: chronic constriction injury
DHEA: dehydroepiandrosterone
FC: fluorocitrate
GFAP: glial fibrillary acidic protein
Iba-1: ionized calcium-binding adapter molecule 1
IL-1β: interleukin-1β
IL-1ra: interleukin-1 receptor antagonist
IL-1R1: interleukin-1 receptor type 1
i.t.: intrathecal
ketoconazole: (±)-cis-1-Acetyl-4-(4-[(2-[2,4-dichlorophenyl]-2-[1H-imidazol-1-ylmethyl]-1,3-dioxolan-4-yl)-methoxy]phenyl)piperazine
MA: mechanical allodynia
NeuN: neuronal nuclei
NMDA: N-methyl-D-aspartate
NP: nucleus proprius
P450c17: cytochrome P450c17
PREG: pregnenolone
PWF: paw withdrawal frequency
SDH: superficial dorsal horn
